# Nationwide genetic testing towards eliminating Lafora disease from Miniature Wirehaired Dachshunds in the United Kingdom

**DOI:** 10.1186/s40575-018-0058-8

**Published:** 2018-03-27

**Authors:** Saija Ahonen, Ian Seath, Clare Rusbridge, Susan Holt, Gill Key, Travis Wang, Peixiang Wang, Berge A. Minassian

**Affiliations:** 1 Program in Genetics and Genome Biology, The Hospital for Sick Children, 555 University Avenue, Toronto, ON M5G 1X8 Canada; 2 Department of Pediatrics, University of Texas Southwestern, 5323 Harry Blvd, Dallas, TX 75390-9063 USA; 3 Dachshund Breed Council, Wrington, North Somerset, UK; 4 Fitzpatrick Referrals Orthopedics and Neurology, Halfway Lane, Eashing, Godalming, Surrey UK; 5 School of Veterinary Medicine, Faculty of Health & Medical Sciences, University of Surrey, Guildford, Surrey UK

**Keywords:** Canine polyglucosan storage disease, Progressive myoclonic epilepsy, Miniature Wirehaired Dachshund, DNA testing, Genetic diversity

## Abstract

**Background:**

Canine DNA-testing has become an important tool in purebred dog breeding and many breeders use genetic testing results when planning their breeding strategies. In addition, information obtained from testing of hundreds dogs in one breed gives valuable information about the breed-wide genotype frequency of disease associated allele. Lafora disease is a late onset, recessively inherited genetic disease which is diagnosed in Miniature Wirehaired Dachshunds (MWHD). It is one of the most severe forms of canine epilepsy leading to neurodegeneration and, frequently euthanasia within a few years of diagnosis. Canine Lafora disease is caused by a dodecamer repeat expansion mutation in the *NHLRC1* gene and a DNA test is available to identify homozygous dogs at risk, carriers and dogs free of the mutation.

**Results:**

Blood samples were collected from 733 MWHDs worldwide, mostly of UK origin, for canine Lafora disease testing. Among the tested MWHD population 7.0% were homozygous for the mutation and at risk for Lafora disease. In addition, 234 dogs were heterozygous, indicating a carrier frequency of 31.9% in the tested population. Among the tested MWHDs, the mutant allele frequency was 0.2. In addition, data from the tested dogs over 6 years (2012–2017) indicated that the frequency of the homozygous and carrier dogs has decreased from 10.4% to 2.7% and 41.5% to 25.7%, respectively among MWHDs tested. As a consequence, the frequency of dogs free of the mutation has increased from 48.1% to 71.6%.

**Conclusions:**

This study provides valuable data for the MWHD community and shows that the DNA test is a useful tool for the breeders to prevent occurrence of Lafora disease in MWHDs. DNA testing has, over 6 years, helped to decrease the frequency of carriers and dogs at risk. Additionally, the DNA test can continue to be used to slowly eradicate the disease-causing mutation in the breed. However, this should be done carefully, over time, to avoid further compromising the genetic diversity of the breed. The DNA test also provides a diagnostic tool for veterinarians if they are presented with a dog that shows clinical signs associated with canine Lafora disease.

## Plain English summary

Genetic testing has become an integral tool in purebred dog breeding as more mutations causing hereditary diseases are identified. Many breeders test their dogs prior to breeding to avoid producing dogs at risk of an inherited disease and many breed clubs recommend DNA testing as part of the breed health strategies. As more dogs are tested, information about the mutation frequency becomes available that helps to determine disease risk and prevalence. A mutation causing Lafora disease in dogs has been identified and a genetic test is available to identify dogs at risk, carriers and dogs free of the mutation. Lafora disease is one of the most severe forms of epilepsy to which the Miniature Wirehaired Dachshund (MWHD) is predisposed. Lafora is a late onset recessively inherited disease that leads to neurodegeneration and, often euthanasia within a few years from diagnosis. In this study, we have tested 733 MWHDs from across the world, mostly from the UK, to study the frequency of the disease-causing mutation in MWHDs. This study gives valuable information about the genotype frequencies in the MWHD population. In addition, 6 years of genetic testing shows how a DNA test can be used to reduce the mutation frequency in a dog population. We have shown that, even though the carrier frequency amongst MWHDs is high, it has been slowly decreasing and consequently the frequency of the dogs free of mutation has increased. This shows how beneficial DNA testing is to prevent the breeding of dogs at risk and also how it is possible to adopt an approach to reduce the disease-causing mutation in the MWHD population, while simultaneously recognizing the need to safeguard genetic diversity.

## Background

### Canine Lafora disease

Lafora disease is a fatal, autosomal recessive, progressive, intractable myoclonic epilepsy and is recognized as one of the most aggressive and most severe forms of epilepsy. It has been reported in multiple dog breeds including Basset Hound [1–3], Beagle [4–6], Miniature Wirehaired Dachshund (MWHD) [7, 8], Miniature and Standard Poodle [1, 9], Pointer [10], and Welsh Corgi [4].

Dogs affected with Lafora disease suffer from myoclonus with contractions of the neck and limb muscles. This myoclonus is spontaneous but also triggered by sudden noise, bright light, or visual stimuli and sudden movements close to the dog’s head. Sleep is disturbed by hypnic jerking. [5, 7, 8, 11–13] Affected dogs may also have generalized tonic-clonic or focal seizures [13]. Based on owner descriptions of “panic attacks” it is suspected that affected dogs may suffer frightening visual hallucinations, like human patients [14]. As the disease progresses, the dogs become ataxic, the myoclonus disabling and seizures can become more frequent and severe. In the later stages of the disease, the dogs become blind and develop cerebellar ataxia and increasing signs of cognitive decline with anxiety [5, 7, 8, 11–13]. As the signs become severe the dogs are euthanatized typically a few years from diagnosis [5, 7, 8, 11–13]. The average age of onset is seven years [3, 4, 7, 11, 13, 15].

Lafora disease in dogs is caused by a repeat expansion mutation in the *NHL repeat containing E3 ubiquitin protein ligase 1* (*NHLRC1*) gene [6, 8], a gene known to cause Lafora disease in humans [16]. In humans also, another gene, *laforin* (*EPM2A*) causes the disease, but *EPM2A* has not been associated with it in dogs [17]. Dogs, relative to other species, are predisposed to Lafora. Their genome uniquely contains a dodecamer repeat sequence within the *NHLRC1* gene, which is prone to expansion. This expansion mutation prevents transcription, and, when biallelically present, leads to loss of function, and to Lafora disease [6, 8]. To date, in all Lafora affected dogs in which a mutation has been detected (MWHD [8], Basset Hound [8], Beagle [6]), the mutation has been the *NHLRC1* repeat expansion mutation. As such, this is a species-wide problem that will likely appear spontaneously in many breeds.

The loss of function of the *NHLRC1* leads to abnormal glycogen synthesis. Normally, the glucose storage molecule, glycogen, is soluble. During glycogen synthesis, while glycogen synthase (*GYS1*) extends glucan chains through α1–4 linkages, every 6 units added are removed by 1,4-alpha-glucan branching enzyme 1 (*GBE1*) and reattached upstream through an α1–6 bond, growing the molecule radially into a sphere [17]. Precisely how the sphericity of glycogen is ensured remains unknown, though it is clear that this is managed through a tight regulation by laforin of a small amount of covalently bound phosphate on glycogen [18–21]. The malstructured insoluble glycogen (polyglucosans) gradually precipitates over the months and accumulates into Lafora bodies (LB), which cannot be digested by the normal glycogen-digesting enzymes. LB accumulate in the somatodendritic compartments of neurons and clog dendrites [1, 5, 22] leading to severe and rapid neurodegeneration.

### Unraveling the disease genetics benefits purebred dog breeding

In purebred dogs, extensive inbreeding and a use of popular sires has led to a significant increase of inherited diseases in different breeds. However, after mapping of the dog genome [23, 24], the list of identified disease-causing mutations has expanded significantly and genetic tests are offered for multiple disorders in many dog breeds [25]. Dog breeders are increasingly utilizing genetic testing in the breeding of purebred dogs, to reduce the frequency of disease-associated mutations in the population in a controlled manner without decreasing the genetic diversity of the breed. Genetic testing also provides important information about the prevalence of the mutant alleles in the dog population and provides a diagnostic tool for veterinarians.

Dachshunds are purebred dogs that are increasingly popular as pets [26]. The Fédération Cynologique Internationale (FCI) recognizes nine and the UK Kennel Club (KC) Breed Standard defines six types of Dachshunds: three sizes -Rabbit (FCI only), Standard and Miniature and each of these comes in three coat types - smooth, long and wirehaired.

The origins of the Dachshund can be traced back to short-limbed working dogs that could pursue quarry underground, for example badgers, foxes and rabbits. The breed has been used as a sporting dog for several centuries. It has not been proved conclusively but Germany is generally acknowledged to be the country of origin. The breed is primarily owned as a pet but popular activities include showing and working, with the MWHD being particularly noted for its ability to track a scent and hunt vermin. The estimated United Kingdom (UK) population of KC registered Miniature Wirehaired Dachshunds (MWHD) is around 8000 dogs.

After identification of the canine Lafora disease causing mutation in MWHDs in 2005 [8], a genetic test development was initiated in 2010 in collaboration with the UK Wirehaired Dachshund Club [27] and has been offered since 2012 for the breeders and dog owners. The Wirehaired Dachshund Club with the support from the Dachshund Breed Council undertook a program of education for breeders and owners to raise awareness of the disease and the importance of screening dogs before breeding from them. They also raised funds to help further develop the DNA tests and to subsidize a UK-wide screening program.

We have used the data from the canine Lafora DNA test to evaluate the prevalence of the *NHLRC1* dodecamer repeat expansion mutation among the tested MWHD population, mostly of UK origin. The disease has late-onset clinical signs and the dog is clinically normal during breeding age so genetic testing provides an important tool to avoid breeding dogs at risk of Lafora. The data from this study is likely to help the MWHD breeders and owners to decrease the mutation frequency in the breed and to further validate the DNA test as a reliable tool to use in planning MWHD breeding. This test can also be used as a tool in veterinary diagnostics if a dog is showing any clinical signs that are associated with canine Lafora disease.

## Methods

A diagnostic DNA test for canine Lafora disease was developed at the Hospital for Sick Children, Toronto, Canada in collaboration with the UK MWHD breed club. Genetic testing was encouraged amongst UK MWHD owners and breeders [28] and in 2014 became a requirement for UK Kennel Club Assured Breeders of MWHD. Samples from 733 privately owned pet MWHDs, 502 females and 191 males, were collected for the purpose of diagnostic genetic testing for canine Lafora disease to determine the genotype and allele frequencies in the tested population. MWHD owners were made awere of the availability of the test and they send samples of their dogs for testing. The testing set was a random set of dogs from the MWHD population. As genealogical data was not available for all the DNA tested dogs, the allele frequency was calculated based on all dogs. In addition to the DNA samples, information about gender, country of origin and testing year was collected.

The study cohort was divided into UK, North American (United States and Canada), European (including UK) and Australian populations. Dogs were assigned to groups according to country of birth e.g. dogs originating from outside UK were not included in the UK population even if the dogs now resided in UK***.***

In addition, in liaison with the UK Kennel Club, the annual (2012–2017) number of both safe litters not at risk of Lafora disease (clear x clear, carrier x clear, homozygous x clear) and at risk litters (carrier x carrier, carrier x not tested, clear x not tested, not tested x not tested, homozygous x not tested) were calculated.

**Fig. 1:**
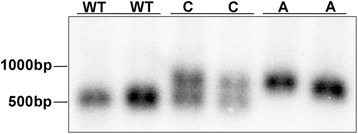
Genotyping is performed using Southern blot. Southern blot is used for genotyping as PCR based methods cannot reliably distinguish the different genotypes due to the type of the mutation. The affected dogs (A) are homozygous for the dodecamer repeat expansion mutation with multiple dodecamer repeats, carrier (C) dogs have the normal and mutated allele and clear dogs (WT) have three copies of the repeat [8]

Genomic DNA was extracted from whole blood in EDTA using phenol-chloroform extraction. The DNA concentration was measured using NanoDrop (Thermo Fisher) and stored in − 20 °C. Genotyping was performed using Southern blot as described by Hajek et al. 2016 [6]. Genomic DNA (10 μg) was digested with DraIII (New England Biolabs #R3510) and EcoRI (New England Biolabs #R0101L) restriction enzymes overnight. Genomic DNA was separated on 1% agarose gel, and nicked with 0.3 M HCl. Before transferring to Hybond-N membrane (Amersham Hybond N+) the DNA was denatured with 0.5 M NaOH + 1.5 M NaCl, and neutralized with 0.2 M Tris (pH 7.4). After pre-hybridization, the membrane was hybridized with a P^32^-labelled DNA fragment specific to canine *NHLRC1* (GeneBank #AY560905.1, CanFam3.1, chr35:16,920,972–16,921,534). After several rounds of washes with 1 M sodium phosphate, 20% SDS, the membrane was exposed onto X-ray film. Normal dogs (WT) have one 563 bp band and affected dogs (A) with the dodecamer expansion have an close to 1000 bp band depending on the number of repeats [8] and heterozygous carriers (C) have one normal and one mutated allele (Fig. 1). Southern blot is used as PCR based methods cannot reliably distinguish different genotypes, specifically heterozygous dogs due to the type of dodecamer repeat expansion.

Statistical analyses were performed using GraphPad Prism version 7.0 (GraphPad Software, La Jolla California USA). Statistical significance between gender and genotype was calculated using Chi-square method. Cochran-Armitage trend test was used to calculate statistical significance for annual genotype frequencies.

## Results

A genetic test for Lafora disease was used to test a total of 733 MWHDs with the owners receiving a report indicating the genotype (clear, carrier, or affected). Within the tested dogs 7.0% (51/733) were homozygous for the *NHLRC1* dodecamer repeat expansion mutation associated with canine Lafora disease [8]. The proportion of carriers was 31.9% (234/733) and the rest of the tested dogs 61.1% (448/733) were clear (Table 1).

**Table 1:** Number and genotype frequency of tested MWHDs and estimation of the mutation allele frequency

	Number of dogs				Genotype frequency (%)			Mutation allele frequency
	Total	Clear	Carrier	Homozygous	Clear	Carrier	Homozygous	
Total	733	448	234	51	61.1	31.9	7.0	0.2
Australia	17	16	1	0	94.1	5.9	0	0.03
Europe^a^	604	363	202	39	60.1	33.4	6.5	0.2
UK only	548	316	193	39	57.7	35.2	7.1	0.2
North-America	61	40	17	4	65.6	27.9	6.6	0.2

^a^including UK

A total of 733 MWHDs were DNA tested in this study. Within the tested dogs 7.0% were homozygous for the *NHLRC1* mutation and at risk for LD and 31.9% of the tested dogs carries the mutation. Similar frequencies were observed among the European population, including dogs of UK origin and in the dataset of dogs originating from UK only. Only small cohorts originated from Australia and North-America

In total, the MWHDs originated from 20 different countries mostly (604/733) from European countries, 61 from North-America (United States and Canada) and 17 from Australia (Table 2). Data for 51 dogs was not available. A total of 548 dogs among the tested dogs were of UK origin and in this population 7.1% (39/548) of the dogs tested homozygous for the mutation, 35.2% (193/548) were carriers and 57.7% (316/548) tested clear (Table 1). The proportion of homozygous dogs among the North-American and European population including UK was 6.6% (4/61) and 6.5% (39/604), respectively (Table 1). The carrier frequency varied from 27.9% (17/61) among the North-American MWHDs to 33.4% (202/604) in the whole European population (including UK) (Table 1). The frequency of the mutated dodecamer repeat allele among all 733 tested MWHDs was 0.2. The same allele frequency was detected among the North-American, European and in the UK population (Table 1).

**Table 2:** The MWHDs originated from 20 different countries

		Number of dogs			Genotype frequency (%)		
Country	Total	Clear	Carrier	Homozygous	Clear	Carrier	Homozygous
Australia	17	16	1	0	94.1	5.9	
Canada	1	1	0	0	100		
Czech Republic	2	1	1	0	50.0	50.0	
Estonia	1	1	0	0	100		
Finland	6	4	2	0	66.7	33.3	
France	3	0	3	0		100	
Germany	3	3	0	0	100		
Hungary	2	1	1	0	50.0	50.0	
Ireland	2	2	0	0	100		
Italy	3	3	0	0	100		
Latvia	2	1	1	0	50.0	50.0	
Norway	1	1	0	0	100		
Poland	2	2	0	0	100		
Russia	20	20	0	0	100		
Serbia	2	2	0	0	100		
Spain	3	3	0	0	100		
Sweden	3	2	1	0	66.7	33.3	
Ukraine	1	1	0	0	100		
United Kingdom	548	316	193	39	57.7	35.2	7.1
United States	60	39	17	4	65	28.3	6.7
Unknown	51	29	14	8	56.9	27.5	15.7
Total	733	448	234	51	61.1	31.9	7.0

Most of the tested dogs were from European countries, but samples were also sent from Australia, Canada and the United States. As the UK Kennel Club has promoted the LD test among the UK MWHD breeders and owners, most of the dogs included in this study originated from the UK

Among all tested MWHDs, 68.5% (502/733) were females compared to 26.1% (191/733) of males. Gender data was not available for 40 dogs. Of the males tested, 66.0% tested clear, compared to 59.4% of females. 28.8% and 5.2% of males were carriers or homozygous, respectively. The same percentages for females were 33.7% carriers and 7.0% homozygous (Table 3). Although more females were tested, no statistically significant difference was detected between gender and genotype (Chi-Square = 2.6, *p* = 0.3).

**Table 3:** Number and percentage of dogs tested based on gender

	Number of dogs	% of total	Clear		Carrier		Homozygous	
			Total	%	Total	%	Total	%
Male	191	26.1	126	66.0	55	28.8	10	5.2
Female	502	68.5	298	59.4	169	33.7	35	7.0
Unknown	40	5.5	24	60.0	10	25.0	6	15.0
Total	733		448		234		51	

More females were tested as commonly fever males are used for breeding. Among the genotype groups, clear, carrier and homozygous, significantly more females were included compared to the same groups among males

**Fig. 2:**
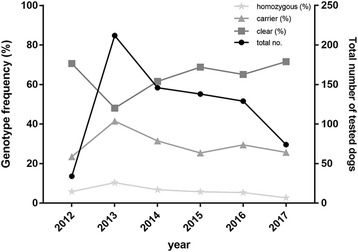
Yearly number of tested dogs and genotype frequencies. The frequency of the carrier and homozygous dogs at risk of Lafora has decreased after a few years of genetic testing. As a consequence the frequency of the clear dogs has increased

The canine Lafora disease test has been available since 2012 when the first dogs were tested to validate the DNA test. Since the test was made available in 2013, approximately 70–200 dogs have been tested yearly (Fig. 2). A decrease in the frequency of carrier dogs can be seen from 41.5% in 2013 to 25.7% in 2017. A similar trend was detected in the frequency of homozygous dogs from 10.4% in 2013 to 2.7% from 2015 to 2017 (Table 4). As a consequence, the frequency of clear dogs has increased from 48.1% in 2013 to 71.6% in 2017 (Table 4) (Cochran-Armitage trend test *p* = 0.0012). Based on the UK Kennel Club data of litter registration data, increase was also detected in the number of safe litters born in UK from 45% in 2012 to 92.1% in 2017 and subsequently a decrease in the unsafe litters from 55% to 7.9% between 2012 and 2017 (Table 5).

**Table 4:** Yearly number and genotype frequency of the tested MWHDs

	Genotype frequency (%)					
Year	2012	2013	2014	2015	2016	2017
Clear	70.6	48.1	61.6	68.8	65.1	71.6
Carrier	23.5	41.5	31.5	25.4	29.5	25.7
Homozygous	5.9	10.4	6.8	5.8	5.4	2.7
Total no. of dogs	34	212	146	138	129	74

LD DNA test was developed in 2012 and after initial validation of the test in a small cohort of MWHDs the test has been offered since 2013. After a few years of genetic testing a decrease in the frequency of homozygous and carrier dogs can be observed and as a consequence the frequency of clear dogs has increased over the years. The slow decrease indicates that carrier dogs are kept as part of the breeding program to avoid compromising the genetic diversity of MWHDs

**Table 5:** Yearly frequency of safe and at risk litter born in UK

Year	2012	2013	2014	2015	2016	2017
Safe litter	45.0	48.6	68.9	84.5	91.8	92.1
At risk litter	55.0	51.4	31.1	15.5	8.2	7.9
Total no. of litters	40	175	183	193	183	164

An increase was detected in the number of safe litters born in UK between 2012 and 2017. Subsequently there is a decrease in the number of unsafe litters indicating that breeders are using the genetic information obtained for the Lafora DNA test

## Discussion

### Practical actions to reduce the Lafora risk in MWHDs by the breed club

Lafora disease can occur in any dog breed due to the type of identified dodecamer repeat mutation associated with the disease [8]. However in the Basset Hound, Beagle and Miniature Wirehaired Dachshund the same dodecamer repeat has been identified as causative in all three breeds [6, 8].

The problem of Lafora in MWHDs was recognized by the UK breed club and could be addressed because there is a series of canine organizations concerned with safeguarding and protecting the health and welfare of dogs. The development of a DNA test was started in collaboration with the researchers and since the Lafora disease DNA test was developed, the UK Kennel Club has maintained a publicly available database of the Lafora test results from dogs that participated in the UK Kennel Club DNA screening scheme [29].

Each quarter, the UK Kennel Club publishes details of new litters that have been bred so the Breed Council has been able to ascertain the DNA test status of the parents of each litter. That has been a key input to the Breed Council’s quarterly communication updates to breeders and owners on the success or otherwise of the screening program. Informing people about the number of puppies likely to be affected was a powerful message to increase DNA screening in MWHDs and increase the number of safe litters.

As expected, some MWHD breeders have chosen not to participate because of the decision to publish the results. However, it was considered important to be open and transparent about the extent of the problem in the breed. Similar way as hip, elbow and eye examination results are publicly available [30]. In addition, communication of the need for screening was directed at owners and potential owners, as well as at breeders. This helped to create “demand side” pressure for Lafora-screened litters. Publishing the data on the proportion of unaffected and “at risk” litters (carrier x carrier, carrier x not tested, clear x not tested, not tested x not tested, homozygous x not tested) every quarter from the Breed Records Supplement provided further evidence of progress and is a good way to recognize what is being achieved and to support participating breeders.

An important aspect of avoiding or anticipating unintended consequences that might arise from implementing a screening program was to understand the systemic impact of the decision. With the Lafora DNA test, some breeders denied that there was a problem in MWHDs, despite the evidence from test results. Another response was to challenge the validity and reliability of the test. However, DNA testing of 733 MWHDs provides evidence-based data showing reliably the genotype frequencies in the population. Interestingly, a number of MWHD pet owners started campaigning for wider adoption of the test by breeders, with their experience of what it was like living with a Lafora disease affected dog.

This particular study of 733 MWHDs provides evidence to evaluate the genotype and allele frequency of the Lafora disease-causing mutation in the MWHD population, especially in the UK population with 548 tested dogs. The high number of tested dogs gives reliable information about the genotype frequencies among the MWHD population included in the study. However, the data represents allele and genotype frequencies over six years of testing and may not be representative of a current situation among MWHDs and as it is a random set of dogs from the whole MWHD population there might be a biased towards clear dogs if owners suspected their dogs being carriers or homozygous dogs and did not want to participate. On the other hand, the data does indicate that the carrier frequency is high and it is unlike that there would have been a dramatic drop among the current population or in a bigger testing population. In addition, the dataset represents mostly dogs of UK origin and genotype and allele frequencies cannot be generalized to the MWHD population worldwide. Genotype frequencies cannot be estimated in countries where testing numbers are low and do not provide statistical confidence. In addition, it is possible that breeders who suspect that they have carrier dogs or dogs at risk of Lafora disease are more likely to screen their dogs. Although the genotype and allele frequencies are the similar across Europe and North-America more samples evenly across different countries should be included to confirm this. In addition, more females were included in DNA testing as proportionally fewer males with desired morphological and genetic features are used for dog breeding. However, no significant difference between gender and genotype was observed in this data set.

In 2014, the UK Kennel Club approved the Lafora DNA test as a “required” test for breeders who were members of its Assured Breeder Scheme [31]. Test results are added to the dog’s registration details, triggering publication of results in the next Breed Records Supplement, any new registration certificate, on certificates of any future progeny, and on the Kennel Club Health Test Results website. The Breed Council was able to use this approval to reinforce further the use of the screening program by Breed Club members who are expected to comply with its Code of Ethics [32].

### DNA testing affects the breeding programs

Using the DNA test, Lafora disease in MWHD could be avoided by testing all dogs used for breeding and mating of carriers only with clear dogs. One advice for breeding would be to breed quality carriers to clear-testing dogs and replace quality carrier parents with quality clear-tested offspring for breeding. This would ensure the eradication of Lafora disease in purebred MWDH. Carriers should be kept as part of the breeding population as other recessive alleles may become more frequent and the genetic diversity of the breed might be compromised if the carrier dogs are removed from the breeding pool too fast. This is particularly important in MWHDs that have a Lafora disease carrier frequency of 31.9% among all the tested dogs and 35.2% in the UK population. Also, Lafora disease is not only genetic disorder in MWHD, other inherited diseases include, intervertebral disc disease (IVDD), ocular disorders, kidney disease and osteogenesis imperfecta [33–37]. The prevalence of IVDD in MWHDs in 17.7% [38] which is significantly higher than the frequency of 7.1% of homozygous *NHLRC1* mutation associated with Lafora disease presented here. However, there has not been a genetic test available for IVDD until recently [39] which is likely to reduce the IVDD prevalence in MWHDs. It is possible that IVDD prevention has higher priority in breeders’ minds than prevention of Lafora disease but as genetic testing has become routinely used tool in dog breeding both test are likely to be used in MWHD breeding to prevent both diseases. The estimated effective population size of MWHDs in UK is 110, which could be considered to be a relatively safe level in order to maintain a viable population [40] and fortunately a number of dogs have been imported with non-UK bloodlines, so there is a wider choice of dogs to use for breeding.

The Wire Haired Dachshund Club (WHDC) decreed that a condition of using the subsidized screening program was that Lafora-affected dogs be excluded from future breeding programs to reduce the number of carriers in the population and because of possible risk to the individual dog as it was not known if breeding of a genetically affected female dog may negatively affect the disease course.

The MWHD population in the UK provides an interesting test population where the genetic test is used to screen the dogs used for breeding. However, even with the genetic test, there has only been a slight decrease in the frequency homozygous and carrier dogs. This indicates that even with the genetic testing the Lafora disease-causing mutation remains at a high frequency in the population. A limitation of this study is that only genetically tested dogs were included in this study and dogs clear based on parental genotypes were not, which might have had an effect to the observer decrease in the genotype frequencies. On the other hand, it is better for breed-wide health as a marked decrease in the genotype would suggest reduced breed genetic diversity. Over several more years of DNA testing, with the same slowly decreasing trend of frequency of carrier and homozygous dogs, the Lafora disease-causing mutation might be eradicated from the MWHD population. We also observed a decline in the number of dogs tested annually possibly due to availability of hereditary clear dogs for breeding and the owners do not need to test their dogs as the genotypes can be estimated based on the parental genotypes. In addition, the number of safe litters has also increase during the years the Lafora test has been available indicating that the test if used as a tool for breeding and breeders use either the test or the hereditary clear dogs for breeding to increase the number of safe litters.

### The international dimension

MWHDs are owned and bred worldwide and the WHDC recognized that the Lafora problem was unlikely to be limited to UK-owned dogs. After development of the DNA test, breed clubs outside UK were encouraged to recommend to their members to test dogs. Consequently, screening is now being carried out by many breeders across the world, notably in the USA, Canada and Australia.

That MWHDs are owned and bred internationally also provides opportunities for reducing the risk of Lafora disease. The mutation was largely identified in UK-bred dogs, and most dogs outside UK were found to be free of the mutation. Importing these dogs and breeding them with UK dogs would enable breeders to reduce the risk of Lafora disease. Conversely, overseas breeders that previously or are currently importing from the UK needed to be aware of the risks they were facing if they are using or have used untested dogs in their breeding programs.

### Other size and coat varieties of Dachshunds do not have Lafora disease-causing mutation

Interestingly the MWHD is the only Dachshund variety in which Lafora disease is known to occur, although there are 8 other varieties under the Fédération Cynologique Internationale (FCI) breed standards based on size and coat type. In some countries the cross-breeding of different coats and sizes is permitted. This practice also happened in the UK until the mid-1970s when it was decided that puppies from such litters would no longer be registered. Outside the UK, this means that it would be feasible for the Lafora disease-causing mutation to be introduced into one of the other varieties of Dachshund where it could become widespread in the population if no screening takes place. In the UK, because of the pre-1970s practice of cross-variety breeding, smooth-coated puppies are occasionally born in litters from two wire-coated parents. This is known as a “recessive coat”; the gene for the smooth coat being recessive to the gene for the wire coat. In 2016, the UK KC decided to amend the registration regulations, allowing the registration of Dachshunds born with a recessive coat type. The Dachshund Breed Council expressed concern that this could lead to Lafora disease entering the gene pool of other varieties. After careful consideration, the Kennel Club Board approved a recommendation from their Dog Health Group that the progeny of any Dachshund from two Miniature Wirehaired Dachshund parents, registered as a different coat type to their parents, must have a coat-type DNA test as a condition of registration, and will be endorsed by the Kennel Club (progeny not eligible for registration). The endorsement will be removed only should a clear Lafora test result be produced or if both parents are either tested as clear, or shown to be clear based on their pedigree.

## Conclusions

Our data provides valuable information about the genotype frequency of the Lafora disease associated mutation and indicates a high carrier frequency in the MWHD population. Years of genetic testing has resulted in reduction of Lafora disease carriers and homozygous dogs. Our study validates the canine Lafora disease DNA test as a valuable tool to use as part of the MWHD breeding scheme.

## Abbreviations

*EPM2A*: *Laforin*
FCI: Fédération Cynologique Internationale
*GBE1*: *1,4-alpha-glucan branching enzyme 1*
*GYS1*: *glycogen synthase 1*
HWE: Hardy-Weinberg equilibrium
IVDD: intervertebral disc disease
KC: Kennel Club
LB: lafora body
MWHD: Miniature Wirehaired Dachshund
*NHLRC1*: *NHL repeat containing E3 ubiquitin protein ligase 1*
UK: United Kingdom
WHDC: Wire Haired Dachshund Club
