# Correction to: Nationwide genetic testing towards eliminating Lafora disease from Miniature Wirehaired Dachshunds in the United Kingdom

**DOI:** 10.1186/s40575-021-00104-3

**Published:** 2021-12-27

**Authors:** Saija Ahonen, Ian Seath, Clare Rusbridge, Susan Holt, Gill Key, Travis Wang, Peixiang Wang, Berge A. Minassian

**Affiliations:** 1 Program in Genetics and Genome Biology, The Hospital for Sick Children, 555 University Avenue, Toronto, ON M5G 1X8 Canada; 2 Department of Pediatrics, University of Texas Southwestern, 5323 Harry Blvd, Dallas, TX 75390–9063 USA; 3 Dachshund Breed Council, Wrington, North Somerset UK; 4 Fitzpatrick Referrals Orthopedics and Neurology, Halfway Lane, Eashing, Godalming, Surrey, UK; 5 School of Veterinary Medicine, Faculty of Health & Medical Sciences, University of Surrey, Guildford, Surrey, UK

**Correction to: Canine Genet Epidemiol 5, 2 (2018)**

**https://doi.org/10.1186/s40575-018-0058-8**

Following the publication of the original article [1], the authors identified an error in Fig. 1. The correct figure is given below and the original article has been corrected.

**Fig. 1:**
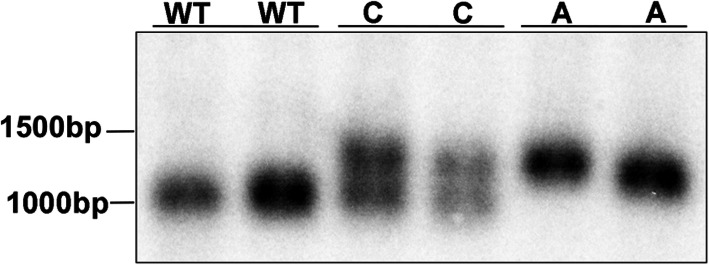
Genotyping is performed using Southern blot. Southern blot is used for genotyping as PCR based methods cannot reliably distinguish the different genotypes due to the type of the mutation. The affected dogs (**a**) are homozygous for the dodecamer repeat expansion mutation with multiple dodecamer repeats, carrier (**c**) dogs have the normal and mutated allele and clear dogs (WT) have three copies of the repeat [8]
